# Serum IgG Is Associated With Risk of Melanoma in the Swedish AMORIS Study

**DOI:** 10.3389/fonc.2019.01095

**Published:** 2019-10-29

**Authors:** Anna Kessler, Sam Sollie, Sophia N. Karagiannis, Goran Walldius, Niklas Hammar, Mieke Van Hemelrijck

**Affiliations:** ^1^Translational Oncology & Urology Research, School of Cancer and Pharmaceutical Sciences, King's College London, London, United Kingdom; ^2^School of Basic and Medical Biosciences, St John's Institute of Dermatology, King's College London, Guy's Hospital, London, United Kingdom; ^3^Unit of Cardiovascular Epidemiology, Institute of Environmental Medicine, Karolinska Institutet, Stockholm, Sweden; ^4^Unit of Epidemiology, Institute of Environmental Medicine, Karolinska Institutet, Stockholm, Sweden

**Keywords:** melanoma, AMORIS cohort, immunoglobin, IgG, humoral immunity, melanoma risk

## Abstract

**Background:** Relatively little is known about the role of the humoral immune system in melanoma. Tumor infiltrating B cells in melanoma patients have been associated with increased T cell activation in tumors as well as improved patient survival. Immunoglobulins may play an important part in the anti-tumor immune response. We hypothesized that increased levels of pre-diagnostic serum Ig may be protective against melanoma development. Hence, we evaluated associations between pre-diagnostic serum markers of the immunoglobulin A (IgA), IgG and IgM, and risk of developing melanoma in the Swedish Apolipoprotein-related MORtality RISk (AMORIS) study.

**Methods:** Study participants aged ≥20 years with baseline measurements of IgG, IgA and IgM taken between 1985 and 1996 were selected (*n* = 29,876). All individuals were free from melanoma at baseline and 162 study participants developed melanoma during follow up. Cox proportional hazards regression was carried out for medical cut-offs of IgA, IgG, and IgM.

**Results:** Compared to the reference level of 6.10–14.99 g/l, we observed a positive but not significant association with risk of melanoma for those with IgG levels <6.10 g/L [HR: 1.05 (95% CI 0.39–2.86)] and an inverse association for those with IgG levels ≥15.00 g/L [HR: 0.60 (95% CI 0.34–1.05); *P*_trend_ = 0.08]. No associations with serum IgA or IgM were identified.

**Conclusions:** The humoral response might provide a protective role against the development of melanoma, mediated through IgG. Further research is needed to characterize this response which may be exploitable for development of future therapies.

## Introduction

Since the 1970s, the incidence of cutaneous melanoma has been rapidly increasing (1). Despite recent advances in targeted therapies and immunotherapy, 10-years survival of stage II melanoma is 50% compared to 92% for stage I melanoma. One year survival alone for stage IV melanoma is 33–62% (2). Early diagnosis, therefore remains one of the greatest challenges of melanoma management.

A growing body of evidence has revealed the immunogenicity of melanoma; however, the majority of research has been directed at the role of T cells in melanoma, and there is relatively little data on the humoral immune system and melanoma. Tumor-infiltrating B cells (TIBs) have been identified in melanoma samples and are associated with improved prognosis (3–5). The density of TIBs has been correlated with increased number of activated T cells (3). A local anti-tumoral humoral response, therefore exists. Melanoma-specific B cells have also been identified in the circulation (4) as well as the presence of a melanoma specific auto-antibody signature (6). Conversely, certain studies have revealed immunomodulatory mechanisms that support a Th2-biased immune response associated with reduced mature B cell responses and production of immunoglobulin isotypes, such as IgG4, in tumor microenvironments and the circulation of patients with melanoma (7–10). These re-educated humoral responses are thought to participate in cancer-associated inflammation and may moderate the potency of otherwise cytotoxic antibodies, to prevent immune-driven elimination of melanoma and may even negatively affect prognosis (7, 9, 11, 12). These seemingly opposing roles of the humoral immune system may suggest that an adaptive humoral response may protect from melanoma growth and may be repolarized toward a regulatory state as part of melanoma-associated immune suppression. Toward elucidating these disparate host-protective vs. tumor-promoting functions, the current study aimed to evaluate associations between pre-diagnostic serum markers of the humoral immune system, immunoglobulin G (IgG), IgA and IgM, and risk of melanoma in the prospective Swedish Apolipoprotein-related MORtality RISk (AMORIS) cohort study.

## Methods

### Study Population and Data Collection

The Swedish Apolipoprotein-related MORtality RISk cohort (AMORIS) includes blood and urine samples from 812,073 Swedish residents, predominantly from Stockholm county, collected and analyzed from 1985 to 1996. Laboratory analyses were performed at the Central Automation Laboratory (CALAB), Stockholm. Study subjects were younger than 20 to older than 80 years old and had blood or urine samples collected as part of routine health checks or outpatient testing (13). A more detailed description of the AMORIS cohort is given elsewhere (14–17).

In addition to the laboratory analyses, the AMORIS cohort includes information from 24 different Swedish national health registers, quality of care registers, socio-economic survey data, health questionnaires and biomedical data from a number of research cohorts. Specifically, for the current study we focused on information included the National Cancer Register, the Patient Register, the Cause of death Register, and consecutive Swedish Censuses during 1970–1990 by using the Swedish 10-digit personal identity number (13). This study was compliant with the Declaration of Helsinki and was approved by the Ethics Review Board of the Karolinska Institute.

The outcome investigated in this study was the risk of developing melanoma (ICD 7 code 190) as registered by the National Cancer Register. We restricted our study population to individuals aged 20 years or older, and excluded any individuals diagnosed with melanoma at baseline. Furthermore, all subjects were required to have a baseline measurement of IgA, IgG, and IgM measured at the same time point between 1985 and 1996. If a participant had multiple measurements of an immunoglobulin, the first measurement was included in the study (*n* = 29,876). Follow-up time was defined as time from baseline measurement until date of cancer diagnosis, death, emigration, or end of the study (31st of December 2002), whichever occurred first.

The following information was obtained from the AMORIS study: serum IgA (g/L), IgG (g/L) and IgM (g/L), time of year Ig samples were taken, age at diagnosis, and gender. The quantitative determination of IgA, IgG and IgM were done with a turbidimetric determination with reagents (DAKO—Glostrup, Denmark) using a HITACHI 911 automatic analyser (Boehringer—Mannheim, Germany) with a coefficient of variation <5% (IgA), ≤ 5% (IgG), and ≤ 7% (IgM) (18–20). Information on socio-economic status (SES), education, day light, and Charlson Comorbidity Index (CCI) was also included. The dichotomous variable daylight was defined as the time of year Ig blood samples were taken when there was ≥16 or <16 h of daylight in the Stockholm area, so that the effect of sun exposure on serum Ig levels could be adjusted for.

### Data Analyses

The risk of melanoma was estimated using multivariate Cox proportional hazards regression for medical cut-offs used in the CALAB laboratory of IgA (<0.70, 0.70–3.65, ≥3.66 g/L) and IgG (<6.10, 6.10–14.99, ≥15.00 g/L) (18–21). The medical cut-offs used by CALAB for IgM (<0.39, 0.39–2.08, ≥2.08 g/L) were not used in the analysis due to the small number of participants with high levels of IgM. Instead we have dichotomized IgM as <1.40 and ≥1.40 g/L proposed by the normal laboratory values for blood, plasma and serum from the MSD manual (22). The assumption of proportionality was checked using the Schoenfield residuals and there was no violation. Cox proportional hazards regression models were adjusted for age, gender, education, CCI, and daylight. A test for trend was conducted by using assignment to medical cut-offs as an ordinal scale. To assess reverse causation, a sensitivity analysis was conducted in which subjects with a follow-up time <1 and <3 years were removed.

Stratified analyses for age (<55, ≥55 years) and gender (male, female) were performed for the association between IgG and risk of melanoma. A *P*-value for interaction was also calculated.

Finally, restrictive Cubic Spline (RCS) function was used to graphically display the hazard ratios representing the dose-response relationship between IgG levels and the risk of melanoma. This analysis was performed using the RCS_RegSAS Macro created by Desquibet and Mariotti (23). Knots at the 5th, 50th, and 95th percentiles were used as per the RCS_RegSAS Macro (23, 24).

All statistical analyses were conducted with Statistical Analysis Systems (SAS) release 9.4 (SAS Institute, Cary, NC).

## Results

The characteristics of study participants are displayed in Table 1. The mean follow-up time was 15.3 years, during which 162 participants developed melanoma. The mean age at measurement in participants who later developed melanoma was higher (55.6) than in participants without melanoma (50.8). In subjects with a diagnosis of melanoma during follow-up, there were more women than men (58.64 vs. 41.36%).

**Table 1:** Descriptive statistics of study population.

	**Melanoma** ***N* = 162** ***n* (%)**	**No melanoma** ***N* = 29,714** ***n* (%)**
**Mean age (SD)**	55.6 (14.92)	50.8 (16.25)
<55	73 (45.06)	18,545 (62.41)
≥55	89 (54.94)	11,169 (37.59)
**Gender**		
Male	67 (41.36)	10,819 (36.41)
Female	95 (58.64)	18,895 (63.59)
**SES**		
Unclassified/Missing	18 (11.11)	5,669 (19.08)
Low	63 (38.89)	12,727 (42.83)
High	81 (50.00)	11,318 (38.09)
**Education**		
Missing	6 (3.70)	1,659 (5.58)
Low	38 (23.46)	7,874 (26.50)
Middle	71 (43.83)	12,538 (42.20)
High	47 (29.01)	7,643 (25.72)
**Charlson comorbidity index**		
0	132 (81.48)	26,124 (87.92)
1	20 (12.35)	2,346 (7.90)
2	6 (3.70)	695 (2.34)
3+	4 (2.47)	549 (1.85)
**Mean follow-up time (years) (SD)**	9.9 (5.43)	15.3 (4.75)
**IgG (g/L)**		
Mean (SD)	10.76 (3.21)	11.41 (3.36)
<6.10 g/L	4 (2.47)	557 (1.87)
6.10–14.99 g/L	144 (88.89)	25,435 (85.60)
≥15.00 g/L	14 (8.64)	3,722 (12.53)
**IgA (g/L)**		
Mean (SD)	2.42 (1.20)	2.45 (1.33)
<0.70 g/L	4 (2.48)	635 (2.14)
0.70–3.65 g/L	133 (82.61)	24,487 (82.49)
≥3.66 g/L	24 (14.91)	4,564 (15.37)
**IgM (g/L)**		
Mean (SD)	1.10 (0.61)	1.26 (0.95)
<1.40 g/L	116 (71.60)	20,276 (68.24)
≥1.40 g/L	46 (28.40)	9,438 (31.76)
**IgE (kU/L)**		
Mean (SD)	149.46 (320.73)	132.14 (414.72)
<100 kU/L	11 (6.79)	1,758 (5.92)
≥100 kU/L	2 (1.23)	657 (2.21)
Missing	149 (92.0)	27,299 (91.9)

Multivariate Cox regression (adjusted for age, sex, education, CCI, and daylight) for the association between Ig and risk of melanoma revealed, compared to the IgG reference level of 6.10–14.99 g/l, a positive association with risk of melanoma for those with IgG levels <6.10 g/L [HR: 1.05 (95% CI 0.39–2.86)] and an inverse association for those with IgG levels ≥15.00 g/L [HR: 0.60 (95% CI 0.34–1.05); *P*_trend_ = 0.08]; although this was non-significant. No associations were found with IgA or IgM levels (Table 2).

**Table 2:** Hazard ratio (HR) for risk of melanoma with 95% confidence intervals (CI) using Cox proportional hazards model.

	**Melanoma/Total** ***N***	**Hazard ratio^a^** **(95% CI)**
**IgG (g/L)**		
<6.10 g/L	4/561	1.05 (0.39–2.86)
6.10–14.99 g/L	144/25,579	1.00 (ref)
≥15.00 g/L	14/3,736	0.60 (0.34–1.05)
*P*-value for trend		0.08
**IgA (g/L)**		
<0.70 g/L	4/639	1.11 (0.41–3.00)
0.70–3.65 g/L	133/24,620	1.00 (ref)
≥3.66 g/L	24/4,588	0.79 (0.50–1.23)
*P*-value for trend		0.29
**IgM (g/L)**		
<1.40 g/L	116/20,392	1.00 (ref)
≥1.40 g/L	46/9,484	0.93 (0.66–1.31)

a *Adjusted for age, gender, education, CCI, and daylight*.

A sensitivity analysis, to assess reverse causation by excluding those with follow-up time <1 and <3 years, did not affect the above findings (Supplemental Table 1).

No effect modification by age or gender on the association between IgG and the risk of melanoma was observed (Supplemental Tables 2, 3).

**Figure 1:**
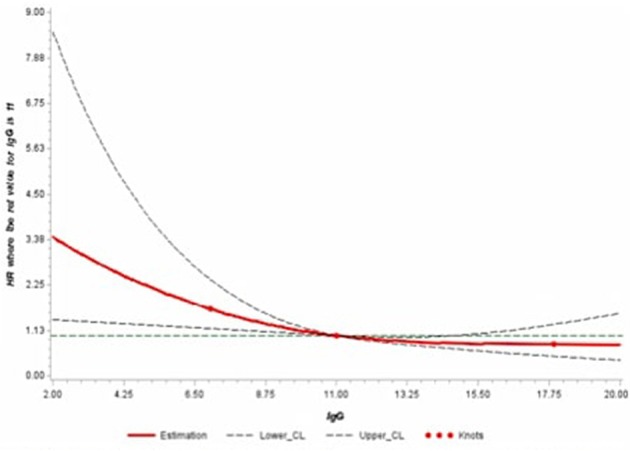
Adjusted dose-response association between serum levels of IgG and risk of melanoma using restrictive cubic splines.

Finally, we further modeled the potential association between serum IgG and the risk of developing melanoma through a dose-response curve with restrictive cubic splines (Figure 1). The direction of the hazard ratios observed in Table 2 was consistent with the shape of the curve.

## Discussion

In this study, evidence for an inverse association between serum IgG levels and the risk of developing melanoma was found in the AMORIS cohort. No associations were found between serum IgA and IgM and the risk of melanoma.

To our knowledge, this is the first prospective cohort study to report the relationship between pre-diagnostic serum IgG and risk of melanoma development. Our findings of a consistent inverse, though not yet statistically significant, association between IgG and risk of development of melanoma suggest that the humoral immune system may play a protective role before the onset of melanoma. This could be consistent with induction of classical immunity and possible protective immune surveillance functions eliminating cancer cells before tumors are able to develop.

Huang et al. report a strong IgG antibody response in sera from melanoma-associated antigens in melanoma patients compared to controls (25). Melanoma-specific IgG antibodies secreted by B cells derived from melanoma patients' peripheral blood, capable of antibody dependent cellular cytotoxicity have also been identified (4). Furthermore, circulating B cells in melanoma patients can express cutaneous leucocyte-associated antigen (CLA), which allows B-cells in the circulation to be recruited to skin (5). TIBs present in melanoma samples are capable of activating T- cells and are associated with improved patient outcomes (5, 8).

Although still speculative, it is conceivable that pre-existing high levels of IgG confer a protective role to development of melanoma through increased immunosurveillance, by mechanisms, such as, antibody dependent cellular cytotoxicity, T cell activation and recruitment of B cells to the skin from the circulation in response to melanoma antigens (3–5, 8).

On the other hand, melanoma tumor-associated immune escape mechanisms may be associated with supporting a regulatory humoral immune state, including expression of immunoglobulin isotypes, such as IgG4 with low effector function potency, meaning the dominance of antibodies less able to engage immune effector cells against cancer (8, 10–12).

No associations were found between serum IgA and IgM and the risk of melanoma. This may be indicative of the quality of the humoral immune response required for effective immune surveillance against melanoma. IgA is primarily associated with mucosal immunity; its main effector function is neutralization of toxins, while it may have weak effector functions and is associated with anti-inflammatory properties (26, 27). IgM is the first line of defense antibody, found on immature B-cells, and as such has relatively low affinity for antigens (28). IgA and IgM, therefore, may not be most effective in protecting against malignant cell transformation.

## Strengths and Limitations

The major strength of this study is the large number of prospective measurements of serum markers of the humoral immune system in the AMORIS cohort, measured at the same clinical laboratory. The database provided complete follow-up for each participant as well as linkage to other registers allowing data collection on cancer status, death or emigration. All participants of the AMORIS study were selected from health checks in non-hospitalized persons. However, any healthy cohort effect would not affect the internal validity of our study. There is no indication that these markers of the humoral immune system were measured due to disease symptoms. Our database contained more women than men, which is likely due to the higher likelihood of assessment of immunoglobulins in women as part of a pregnancy-related health check-up. Sex was treated as a confounder and an effect modifier in the analyses.

The main limitation was the relatively small number of melanoma cases, despite large population size, which makes the significance of results challenging to interpret. Furthermore, there were not sufficient repeated measurements of serum markers of the humoral immune system to verify a trend over time associated with risk of melanoma. Unfortunately, no data on sun exposure was available from the registries used. However, all models were adjusted for daylight by using season as a proxy.

## Conclusion

This is the first prospective cohort study evaluating the association between pre-diagnostic serum markers of the humoral immune system and the risk of melanoma. We observed a consistent inverse, though not statistically significant, association between pre-diagnostic serum levels of IgG and the risk of melanoma. The humoral immune response system may confer protection against melanoma development through immune-surveillance of peripheral blood and cutaneous compartments. IgG antibodies may carry out effective anti-tumor responses via T cell activation and antibody-dependent cellular cytotoxicity. The underlying mechanism has yet to be fully elucidated and further research into the humoral immune response in melanoma in larger melanoma patient cohorts may provide novel therapeutic targets or immunotherapies in the future.

## Data Availability Statement

Publicly available datasets were analyzed in this study. This data can be found here: https://ki.se/en/imm/for-researchers.

## Author Contributions

MV designed the study and supervised the work. AK and SS conducted the data manipulation and analysis. AK wrote the manuscript with input from all authors.

### Conflict of Interest

The authors declare that the research was conducted in the absence of any commercial or financial relationships that could be construed as a potential conflict of interest.
