# Supplementary material for: Serum IgG Is Associated With Risk of Melanoma in the Swedish AMORIS Study
Source: Front Oncol. 2019 Oct 29;9:1095. doi: 10.3389/fonc.2019.01095 (PMC6828930; doi:10.3389/fonc.2019.01095)
Supplement: Supplementary file 1 [file Table_1.DOCX]

**Supplementary Tables**

Table 1. Sensitivity analysis

|  | Follow-up time >1 year | | Follow-up time >3 years | |
| --- | --- | --- | --- | --- |
| IgG (g/L) | Melanoma/ Total N | Hazard Ratio^1^ (95% CI) | Melanoma/ Total N | Hazard Ratio^2^ (95% CI) |
| <6.10 | 4/546 | 1.12 (0.41-3.05) | 4/524 | 1.19 (0.44-3.25) |
| 6.10-14.99 | 134/25,277 | 1.00 (ref) | 126/24,665 | 1.00 (ref) |
| >15.00 | 12/3,638 | 0.55 (0.30-1.01) | 11/3,453 | 0.54 (0.29-1.01) |
| p-value for trend |  | **0.05** |  | **0.048** |
| IgA (g/L) |  |  |  |  |
| <0.70 | 4/632 | 1.21 (0.45-3.27) | 4/611 | 1.28 (0.47-3.48) |
| 0.70-3.65 | 122/24,349 | 1.00 (ref) | 115/23,824 | 1.00 (ref) |
| >3.66 | 23/4,453 | 0.84 (0.53-1.33) | 21/4,180 | 0.82 (0.51-1.33) |
| p-value for trend |  | 0.41 |  | 0.36 |
| IgM (g/L) |  |  |  |  |
| <1.40 | 107/ 20,081 | 1.00 (ref) | 100/19,488 | 1.00 (ref) |
| >1.40 | 43/ 9,380 | 0.93 (0.65-1.34) | 41/9,154 | 0.94 (0.65-1.37) |

^1^ Adjusted for age, gender, education and CCI

^2^ Adjusted for age, gender, education and CCI
