# Supplementary material for: Serum IgG Is Associated With Risk of Melanoma in the Swedish AMORIS Study
Source: Front Oncol. 2019 Oct 29;9:1095. doi: 10.3389/fonc.2019.01095 (PMC6828930; doi:10.3389/fonc.2019.01095)
Supplement: Supplementary file 2 [file Table_2.DOCX]

Supplementary Table

Table 2. Hazard ratios for risk of melanoma with 95% confidence intervals from Cox proportional hazards model.

|  | Age <55 years | | Age >55 years | |
| --- | --- | --- | --- | --- |
|  | Melanoma N/ Total N | Hazard ratio^1^  (95% CI) | Melanoma N/ Total N | Hazard ratio^2^  (95% CI) |
| IgG (g/L) |  |  |  |  |
| <6.10 | 2/ 282 | 1.51 (0.37-6.22) | 2/ 279 | 0.79 (0.19-3.22) |
| 6.10-14.99 | 61/ 16,275 | 1.00 (ref) | 83/ 9,304 | 1.00 (ref) |
| >15.00 | 10/2,061 | 1.07 (0.54-2.14) | 4/1,675 | 0.28 (0.10-0.77) |
| p-value interaction | 0.22 | | | |
| IgA (g/L) |  |  |  |  |
| <0.70 | 2/ 386 | 1.24 (0.30-5.08) | 2/253 | 0.99 (0.24-4.03) |
| 0.70-3.65 | 63/ 16,158 | 1.00 (ref) | 70/ 8,462 | 1.00 (ref) |
| >3.65 | 8/2,059 | 0.78 (0.37-1.67) | 16/ 2,529 | 0.78 (0.45-1.35) |
| p-value interaction | 0.08 | | | |
| IgM (g/L) |  |  |  |  |
| <1.40 | 50/ 12,115 | 1.00 (ref) | 66/ 8,277 | 1.00 (ref) |
| >1.40 | 23/ 6,503 | 0.87 (0.52-1.44) | 23/ 2,981 | 0.98 (0.61-1.58) |
| P-value interaction | 0.77 | | | |

^1^ Exposure variables stratified by age <55, adjusted for age, sex, education and CCI

^2^ Exposure variables stratified by age >55, adjusted for age, sex, education and CCI
