# Supplementary material for: Serum IgG Is Associated With Risk of Melanoma in the Swedish AMORIS Study
Source: Front Oncol. 2019 Oct 29;9:1095. doi: 10.3389/fonc.2019.01095 (PMC6828930; doi:10.3389/fonc.2019.01095)
Supplement: Supplementary file 3 [file Table_3.DOCX]

Supplemental Tables

Table 3. Hazard ratios for risk of melanoma with 95% confidence intervals from Cox proportional hazards model.

|  | Male | | Female | |
| --- | --- | --- | --- | --- |
|  | Melanoma N/ Total N | Hazard ratio^1^  (95% CI) | Melanoma N/ Total N | Hazard ratio^2^  (95% CI) |
| IgG (g/L) |  |  |  |  |
| <6.10 | 2/ 184 | 1.62 (0.40-6.63) | 2/ 377 | 0.78 (0.19-3.19) |
| 6.10-14.99 | 60/ 9,174 | 1.00 (ref) | 84/ 16,405 | 1.00 (ref) |
| >15.00 | 5/ 1,528 | 0.49 (0.19-1.21) | 9/ 2,208 | 0.68 (0.33-1.37) |
| p-value interaction | 0.12 | | | |
| IgA (g/L) |  |  |  |  |
| <0.70 | 1/183 | 0.91 (0.13-6.59) | 3/ 456 | 1.18 (0.37-3.74) |
| 0.70-3.65 | 52/ 8,345 | 1.00 (ref) | 81/ 16,275 | 1.00 (ref) |
| >3.65 | 14/ 2,345 | 0.83 (0.46-1.52) | 10/ 2,243 | 0.72 (0.37-1.41) |
| p-value interaction | 0.06 | | | |
| IgM (g/L) |  |  |  |  |
| <1.40 | 53/ 8,499 | 1.00 (ref) | 63/ 11,893 | 1.00 (ref) |
| >1.40 | 14/ 2,387 | 0.97 (0.54-1.74) | 32/ 7,097 | 0.89 (0.58-1.36) |
| p-value interaction | 0.13 | | | |

^1^ Exposure variables stratified by sex M, adjusted for age, education and CCI

^2^ Exposure variables stratified by sex F, adjusted for age, education and CCI
